# Ethyl Chlorid

**Published:** 1906-07

**Authors:** 


ETHYL CHLORID.—A substance which bids fair to rival chloroform and ether as a general anesthetic, under certain conditions at least, is ethyl chlorid.

This substance is extremely volatile, boiling at from 12.5 to 13 C. (54.5 to 55.4 F.), and it is necessary, therefore, to keep it in sealed tubes. The ends of the tubes are drawn out to fine capillary tubes which are then sealed, or the capillary opening is closed by a metal cap which may be replaced when a part of the contents of the tube has been used. When required for use the glass tip is broken off, or the metal cap unscrewed, when the heat of the hand causes the ethyl chlorid to volatilize, forcing out a fine stream which may be directed against the surface which is to be frozen for local anesthesia, or the stream may supply the vapor for inhalation to produce general anesthesia.

Ethyl chlorid induces anesthesia more rapidly than ether does, and when it is withdrawn the patient recovers more quickly, thus saving an average of some twelve minutes on each operation, hence it is likely to prove useful on the battlefield and in great calamities when a number of operations must be performed with a minimum loss of time.

Figures purporting to give relative degrees of danger for various anesthetics are notoriously unreliable, but it seems probable that ethyl chlorid is less dangerous than chloroform and somewhat more dangerous than ether. Among the objectionable features of ethyl chlorid are increased cost, the explosive character of the vapor, extreme volatility, the accompanying waste of material and the difficulty of administration.—*Journal A. M. A.*

